# Glucagon-Like Peptide-1 Agonist Effects on Weight Loss and Heart Failure Hemodynamics

**DOI:** 10.1016/j.jaccas.2026.110528

**Published:** 2026-09-25

**Authors:** Alberth Alvarado, John Martinez-Ponce, Oskar Ubysz, Anthony Costa, Lina Ataya, Daniel Brancheau

**Affiliations:** a Department of Cardiology, Henry Ford Genesys, Grand Blanc, Michigan, USA; b Department of Internal Medicine, Henry Ford Genesys, Grand Blanc, Michigan, USA

**Keywords:** CardioMEMS, GLP-1 receptor agonists, heart failure, pulmonary artery pressure, weight loss

## Abstract

**Background:**

Optimization of fluid status in heart failure (HF) is challenging. The CardioMEMS system enables real-time pulmonary artery pressure monitoring, whereas glucagon-like peptide-1 (GLP-1) receptor agonists offer metabolic and potential hemodynamic benefits.

**Case Presentation:**

An 82-year-old man with HF (left ventricular ejection fraction: 40%-45%; NYHA functional class III) had recurrent decompensation despite diuretic agents. After CardioMEMS implantation, pressures remained elevated and variable. Initiation of semaglutide led to 20-pound weight loss, improved symptoms, and reduced mean pulmonary artery pressures (10 mm Hg).

**Discussion:**

This case highlights synergy between device-guided hemodynamic monitoring and GLP-1 therapy. Benefits include weight loss, improved congestion, and enhanced functional status, though caution is warranted in reduced-EF populations given mixed trial data.

**Conclusions:**

Device-guided monitoring combined with GLP-1 therapy may enhance HF management. Further studies are needed to confirm safety, long-term outcomes, and applicability across diverse HF populations.

**Take-Home Message:**

Combining CardioMEMS with GLP-1 therapy may improve HF management through tailored, data-driven care, but patient selection remains critical.

## Graphical Abstract

**Figure:**
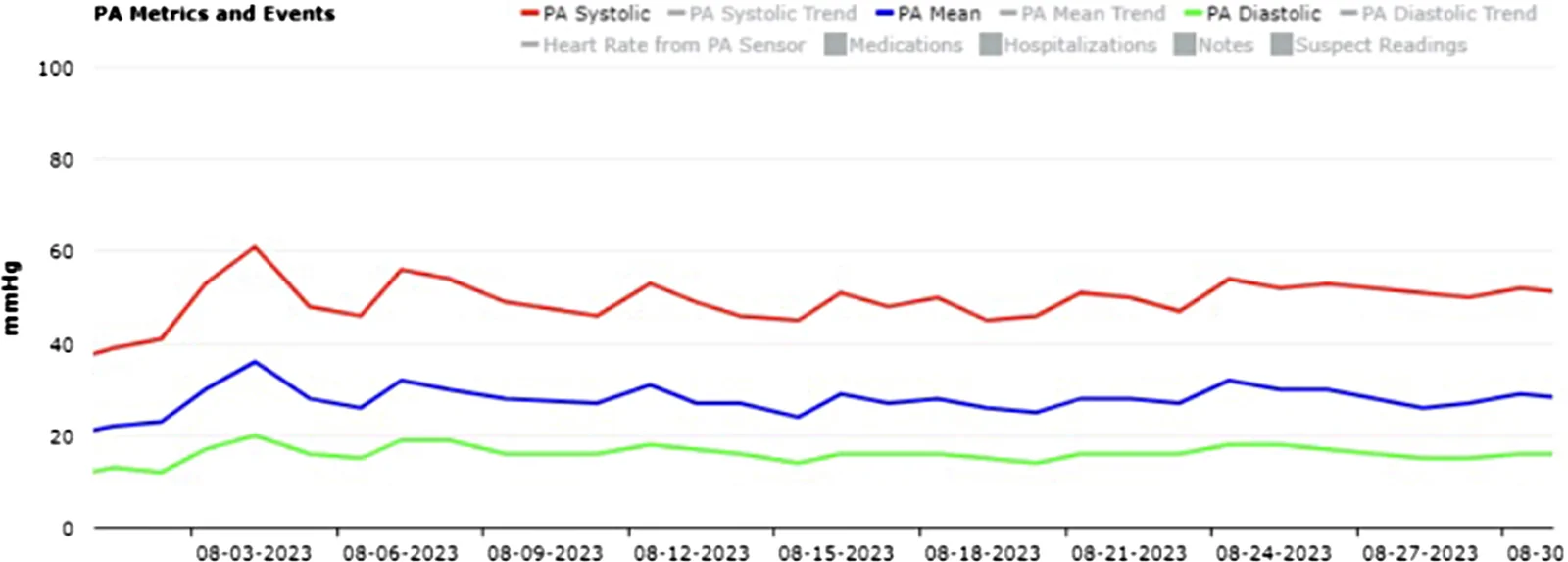

Heart failure (HF), characterized by a spectrum of pathophysiological changes, is a leading cause of morbidity and mortality worldwide. The complexity of HF management demands innovative strategies that integrate device-based monitoring and novel pharmacotherapy.

The CardioMEMS HF System employs a miniaturized, implantable, wireless monitoring sensor placed in the pulmonary artery (PA). This device transmits real-time data on pulmonary artery (PA) pressure (PAP), a crucial hemodynamic parameter in HF management. By enabling daily monitoring of PAP, the CardioMEMS HF System facilitates early detection of hemodynamic changes that often precede clinical symptoms of intravascular fluid congestion. This preemptive approach allows for timely adjustments in therapy, thereby reducing hospitalizations and improving quality of life. The CHAMPION (CardioMEMES Heart Sensor Allows Monitoring of Pressure to Improve Outcomes in NYHA Class III Heart Failure Patients) trial demonstrated a 37% reduction in hospital readmissions among HF patients monitored with the CardioMEMS HF System, underscoring its efficacy and utility in the management of chronic HF.^1^, ^2^, ^3^, ^4^

Glucagon-like peptide 1 receptor agonists (GLP-1 RAs), initially developed for the management of type 2 diabetes, have shown promising effects in HF, regardless of diabetes status. Agents such as semaglutide enhance glucose-dependent insulin secretion while suppressing glucagon release. Beyond glycemic control, GLP-1 RA exhibits cardioprotective properties, including weight loss, improved myocardial energetics, and favorable impacts on blood pressure and lipid profile. The weight loss associated with GLP-1 RA therapy can alleviate the cardiac workload in HF patients, potentially ameliorating symptoms and improving cardiac function.^5^, ^6^, ^7^

## History of Presentation

An 82-year-old man with chronic HF presented with progressive volume overload despite repeated outpatient diuretic agent adjustments. Over approximately 3 months, he gained 40 pounds and developed worsening dyspnea, fluid congestion, and declining exercise tolerance. He had experienced at least one HF hospitalization during the preceding 12 months and was classified as American College of Cardiology stage C with NYHA functional class III symptoms. His clinical deterioration culminated in an 8-day hospitalization for acute decompensated HF requiring intravenous furosemide.

## Past Medical History

The patient had persistent atrial fibrillation and nonischemic cardiomyopathy with a left ventricular ejection fraction (LVEF) of 40%-45%. Prior evaluation showed no documented obstructive coronary artery disease. His chronic heart failure was categorized as American College of Cardiology stage C with NYHA functional class III symptoms. Baseline guideline-directed medical therapy included a beta-blocker and a renin–angiotensin system inhibitor titrated to maximally tolerated doses; further optimization was limited by intermittent hypotension and fluctuations in renal function.

## Differential Diagnosis

The working diagnosis was acute decompensated chronic HF with significant volume overload and elevated right- and left-sided filling pressures. Prior evaluation without documented obstructive coronary artery disease supported the established diagnosis of nonischemic cardiomyopathy rather than an ischemic etiology.

## Investigations

The patient’s known LVEF was 40%-45%. Because of persistent symptoms and difficulty achieving euvolemia, invasive hemodynamic assessment was obtained at the time of CardioMEMS HF System implantation. Initial measurements showed a PA pressure of 45/16 mm Hg with a mean PA pressure of 30 mm Hg, right atrial pressure of 21 mm Hg, right ventricular pressure of 44/17 mm Hg, and mean pulmonary capillary wedge pressure of 24 mm Hg. These findings were consistent with marked volume overload and elevated left-sided filling pressures.

After implantation, daily PA pressure transmission was used for longitudinal assessment. During August 2023, mean PA pressures remained approximately 30-35 mm Hg with substantial variability, and systolic PA pressures were approximately 45-50 mm Hg (Figure 1). After semaglutide initiation, March 2024 recordings showed a more stable hemodynamic profile, with systolic PA pressures approximately 30-35 mm Hg and mean PA pressures approximately 20-25 mm Hg, representing an approximately 10-mm Hg reduction in mean PA pressure (Figure 2).

**Figure 1:**
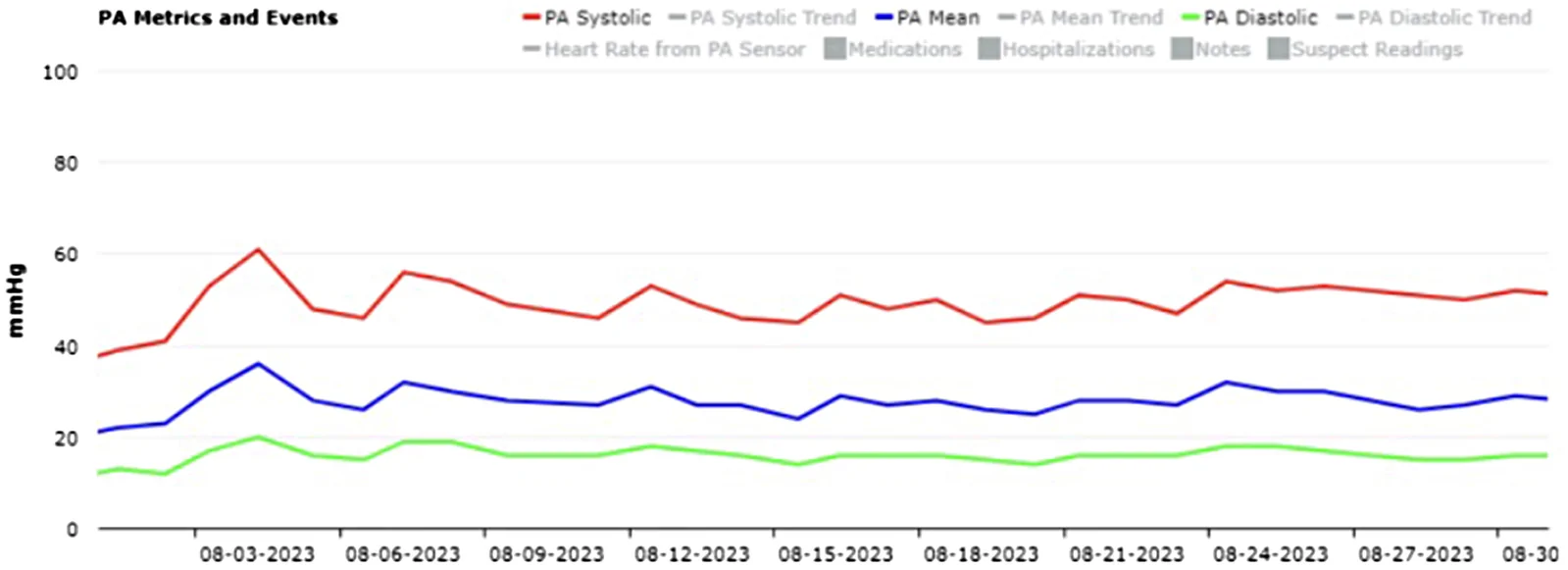
Pulmonary Artery Systolic, Mean, and Diastolic Pressures From August 1, 2023, to August 31, 2024, Showing Large Pressure Fluctuations

**Figure 2:**
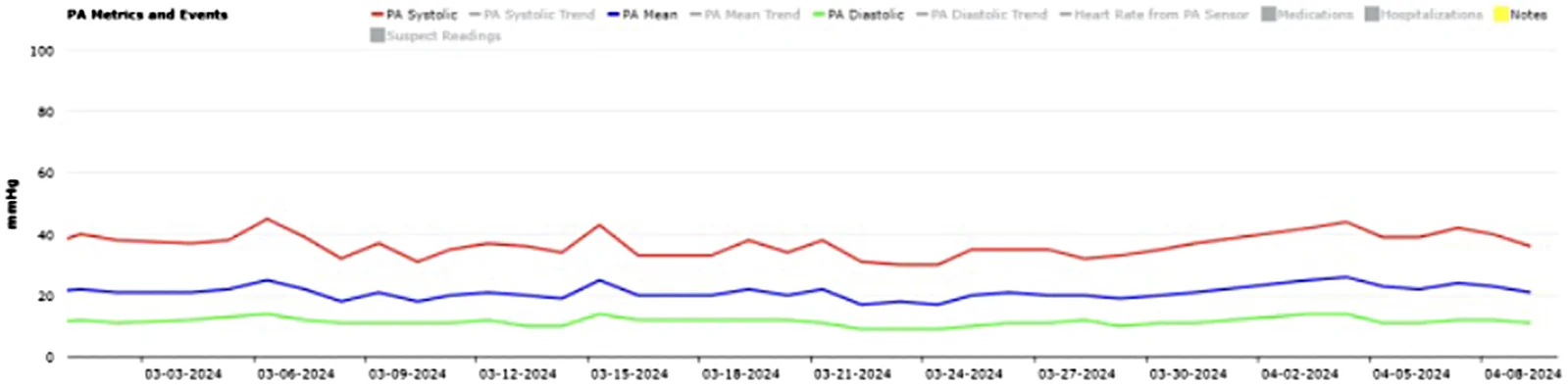
Pulmonary Artery Systolic, Mean, and Diastolic Pressures From March 1, 2024, to April 9, 2024, After the Initiation of Semaglutide

## Management

During the 8-day hospitalization for acute decompensated HF, the patient received aggressive intravenous furosemide. Persistent congestion and difficulty achieving euvolemia prompted escalation to invasive hemodynamic monitoring. He underwent uncomplicated CardioMEMS implantation during the same admission after clinical stabilization. Although implantation is commonly performed in the ambulatory setting after stabilization, the decision to proceed during hospitalization was driven by persistent congestion, NYHA functional class III symptoms, and the recent decompensation.

Following implantation, guideline-directed medical therapy and diuretic agents were continued. PA pressures were reviewed daily with a target mean PA pressure below 25 mm Hg, and diuretic agent doses were individualized according to hemodynamic trends. Persistently elevated pressures and ongoing symptom burden prompted further diuretic agent escalation and close outpatient follow-up.

Several months after CardioMEMS implantation, semaglutide was initiated in the setting of persistently elevated PA pressures and continued symptoms despite medical therapy. As volume status and hemodynamics improved, diuretic agent requirements were gradually reduced. The source study does not specify the patient’s body mass index, diagnosis of obesity, diabetes status, semaglutide dose, or the formal indication for semaglutide; these details should be added if available because they are relevant to interpretation of guideline concordance and generalizability.

## Outcome and Follow-Up

Within 1 month of semaglutide initiation, the patient lost 20 pounds. He reported decreased dyspnea on exertion, improved exercise tolerance, and improved quality of life. CardioMEMS recordings demonstrated an approximately 10-mm Hg reduction in mean PA pressure and less pressure variability, with mean PA pressures decreasing from approximately 30-35 mm Hg before semaglutide to approximately 20-25 mm Hg afterward. Diuretic agent requirements were gradually reduced as the patient’s volume status improved. No procedural complication from CardioMEMS implantation was reported.

## Discussion

The integration of CardioMEMS HF System and GLP-1 RA therapy in HF management reflects evolving clinical practice that focuses on a comprehensive approach to patient care.

CardioMEMS HF System plays a pivotal role in the proactive management of HF. By offering real-time PA pressure data, this technology enables clinicians to preemptively adjust medications and manage fluid status, effectively reducing HF-related hospitalizations. The device's ability to detect subtle changes in hemodynamic parameters enables a nuanced understanding of the patient’s condition, leading to more tailored and effective treatment strategies.^2^

GLP-1 RA, initially used for glycemic control in diabetes, has shown significant promise for cardioprotective effects. These agents promote weight loss, which is beneficial for cardiac function. Weight reduction achieved through GLP-1 RA therapy can improve myocardial energetics by reducing myocardial oxygen demand and enhancing myocardial contraction efficiency. Furthermore, GLP-1 RA may improve endothelial function, thereby contributing to better vascular health and potentially reducing the risk of atherosclerotic disease, as highlighted in studies such as those by Apovian and Gotce^8^ and Rameshrad et al.^5^ In addition to weight reduction, GLP-1 receptor agonists exert several physiological effects that are highly relevant to HF pathophysiology. These agents enhance satiety via central nervous system pathways, thereby reducing caloric intake and sustaining negative energy balance. Beyond metabolic effects, GLP-1 RAs have been shown to promote natriuresis and mild diuresis, likely mediated through inhibition of the sodium-hydrogen exchanger in the proximal tubule. This contributes to reductions in intravascular volume and may directly alleviate congestion. Furthermore, GLP-1 RAs exert favorable neurohormonal effects, including modulation of the sympathetic nervous system and attenuation of maladaptive activation of the renin-angiotensin-aldosterone system. Collectively, these mechanisms may contribute to improved ventricular loading conditions and reduced cardiac wall stress, providing a broader physiological rationale for the observed benefits in patients with HF.^9^

However, recent studies, including the EXSCEL (Exenatide Study of Cardiovascular Event Lowering ) and FIGHT (Functional Impact of GLP-1 for HF Treatment) trials, shed light on the intricate relationship between GLP-1 RA and LVEF. A critical consideration arises in the use of GLP-1 RA, like liraglutide, in patients with HF with reduced ejection fraction. The EXSCEL trial, examining the effects of exenatide in patients with type 2 diabetes, revealed a significant interaction between baseline LVEF and the risk of HF hospitalization. Notably, patients with an LVEF <40% showed an increased risk (OR: 1.70; 95% CI: 1.02-2.83), suggesting a potential concern in this subgroup.^10^

The FIGHT trial further underscored this concern, reporting an odds ratio for HF hospitalization with liraglutide vs placebo of 1.33 (95% CI: 0.83-2.12). These trials collectively suggest that in patients with severe HF with reduced ejection fraction (LVEF <40%), GLP-1 RAs may not be as beneficial and could even increase the risk of adverse outcomes.^10^^,^^11^ Importantly, these findings highlight the heterogeneity of HF phenotypes and suggest that the benefits of GLP-1 RA therapy may be more pronounced in patients with preserved or mildly reduced ejection fraction, particularly when obesity and metabolic dysfunction are central contributors to disease progression.

This brings us to a crucial juncture in our patient’s case: the need for a judicious evaluation of GLP-1 RA therapy, especially considering his cardiac function profile. Although the patient’s initial response to therapy was favorable, with significant weight loss and symptomatic improvement, findings from the EXSCEL and FIGHT trials add complexity and caution.

Weight loss in HF patients, when carefully managed, can have profound effects on cardiac function. Reducing body mass alleviates the strain on the heart, lowering ventricular filling pressures and improving cardiac output. This process can improve myocardial hemodynamics. Additionally, weight loss can enhance endothelial function, leading to improved vascular reactivity and reduced systemic vascular resistance, thereby decreasing afterload and easing cardiac workload.^7^ Moreover, reductions in adiposity are associated with decreased circulating blood volume and lower preload, which may translate into reduced PA pressures, as observed in this case. The combined effects of natriuresis, weight loss, and improved vascular compliance may synergistically contribute to improved congestion profiles and hemodynamic stabilization in patients monitored with CardioMEMS.^12^

The combination of hemodynamic monitoring with the CardioMEMS HF System and GLP-1 RA for weight management represents a synergistic approach to HF care. This strategy addresses both the immediate cardiac needs and the broader metabolic challenges associated with HF, offering a more holistic approach to patient management.

## Conclusions

The case of an 82-year-old man with HF highlights the importance of a multifaceted approach in modern HF treatment. The management of HF is transitioning toward a more dynamic, data-driven, and personalized approach. The integration of CardioMEMS for precise hemodynamic monitoring and the use of GLP-1 RA for metabolic and weight management constitute a multifaceted strategy that addresses both the cardiac and systemic aspects of HF. In our patient, this approach not only improved symptomatic management but also improved overall cardiac function and quality of life.

Future research should focus on exploring the long-term benefits of this combination therapy, particularly in diverse patient populations and in various stages of HF, especially those with LVEF >40%. As cardiologists, embracing these advancements and tailoring them to individual patient profiles will be key in advancing the management of this complex and challenging condition.

**Take-Home Messages**

• Combining CardioMEMS-guided hemodynamic monitoring with glucagon-like peptide-1 therapy may improve heart failure outcomes by promoting weight loss and optimizing fluid management.
• However, careful patient selection is essential, particularly in those with reduced ejection fraction.

**Visual Summary:** Timeline of the Case

Timeline	Events
Day 1 (initial presentation)	82-y-old man with persistent atrial fibrillation, cardiomyopathy (LVEF 40%-45%), ACC stage C chronic heart failure, and NYHA functional class III symptoms after 40-pound weight gain over 3 mo despite multiple diuretic agent adjustments.
Diagnosis/intervention	Admitted for acute decompensated heart failure and treated with intravenous furosemide during an 8-d hospitalization. Because of persistent symptoms and recent decompensation, he was deemed an appropriate candidate for CardioMEMS implantation.
Shortly after stabilization (same admission)	Underwent CardioMEMS Heart Failure System implantation for invasive hemodynamic monitoring. Initial hemodynamics: PA 45/16 mm Hg, mean PA 30 mm Hg, RA 21 mm Hg, RV 44/17 mm Hg, wedge mean 24 mm Hg. Procedure was uncomplicated.
Hospital course	Continued on guideline-directed medical therapy including diuretic agents with close hemodynamic monitoring.
Discharge	Discharged the same day after CardioMEMS implantation following an uncomplicated procedure.
Early follow-up (August 2023)	CardioMEMS reading showed persistently elevated and variable PA pressures with mean PA approximately 30-35 mm Hg and systolic PA 45-50 mm Hg. Clinically, the patient had decreased exercise tolerance and ongoing symptom burden.
Intervention (after persistent elevated pressures)	Semaglutide was initiated.
Late follow-up (March 2024)	After semaglutide initiation, the patient lost 20 pounds within 1 mo, reported improved dyspnea on exertion and exercise tolerance, and CardioMEMS showed improved hemodynamics with around a 10 mm Hg reduction in mean PAP, less variability, systolic PA 30-35 mm Hg, and mean PA 20-25 mm Hg.

ACC = American College of Cardiology; LVEF = left ventricular ejection fraction; PA = pulmonary artery; PAP = pulmonary artery pressure; RA = right atrium; RV = right ventricle.

## Funding Support and Author Disclosures

The authors have reported that they have no relationships relevant to the contents of this paper to disclose.
